# Benzo[a]pyrene-induced immunotoxicity, neurotoxicity, and cardiotoxicity in grass carp (*Ctenopharyngodon idella*)

**DOI:** 10.3389/fimmu.2026.1914171

**Published:** 2026-08-31

**Authors:** Mohamed Hamed, Mervat Naguib, Elsayed S. I. Mohammed, Alhosein Hamada, Abdulaziz R. Alqahtani, Hanem S. Abdel-Tawab, Alaa El-Din H. Sayed

**Affiliations:** 1 Department of Zoology, Faculty of Science, Al-Azhar University (Assiut Branch), Assiut, Egypt; 2 Department of Zoology, Faculty of Science, Assiut University, Assiut, Egypt; 3 Avian Research Center, King Faisal University, Al-Ahsa, Al Hofuf, Saudi Arabia; 4 Department of Arid Land Agriculture, College of Agriculture and Food Sciences, King Faisal University, Al-Ahsa, Al Hofuf, Saudi Arabia; 5 Department of Biology, College of Science, University of Bisha, Bisha, Saudi Arabia; 6 Molecular Biology Research & Studies Institute, Assiut University, Assiut, Egypt

**Keywords:** aquatic toxicology, B(a)P, brain lesions, cardiac alterations, *Ctenopharyngodon idella*, immune dysfunction

## Abstract

**Introduction:**

Benzo[a]pyrene (B[a]P), a widespread polycyclic aromatic hydrocarbon (PAH), is a major aquatic pollutant with known toxic effects. However, its immunotoxicity during early fish development remains poorly understood. This study investigated the immunotoxic, neurotoxic, and cardiotoxic effects of B[a]P in grass carp (*Ctenopharyngodon idella*) Juvenile and evaluated associated neurotoxic and cardiotoxic alterations.

**Methods:**

Grass carp Juvenile were exposed to 1, 10, and 100 μM B[a]P for 14 days. Immune responses were assessed by measuring myeloperoxidase (MPO), nitric oxide (NO), nitroblue tetrazolium (NBT), and immunoglobulin M (IgM). Acetylcholinesterase (AChE) activity was determined as a neurotoxicity marker. Histopathological and histochemical analyses of brain and heart tissues were performed to evaluate tissue damage.

**Results:**

B[a]P exposure significantly increased MPO and NO levels, indicating activation of inflammatory and oxidative responses. In contrast, NBT activity and IgM concentrations were significantly reduced, suggesting suppression of innate and adaptive immunity. AChE activity was markedly inhibited, demonstrating neurotoxicity. Histopathological examination revealed dose-dependent degeneration, necrosis, vascular congestion, and tissue disorganization in cerebellar and cardiac tissues. Histochemical analyses showed alterations in neuronal RNA distribution and changes in cardiac polysaccharide and glycoprotein contents.

**Conclusion:**

B[a]P induces significant immune dysfunction in grass carp Juvenile, characterized by inflammatory activation and suppression of immune defenses. These effects are accompanied by neurotoxic and cardiotoxic damage, highlighting the potential ecological risks of B[a]P contamination in aquatic environments.

## 1 Introduction

Aquatic ecosystems are increasingly exposed to anthropogenic contaminants, including polycyclic aromatic hydrocarbons (PAHs), which are persistent, toxic, and prone to bioaccumulation (1, 2). Among PAHs, benzo[a]pyrene (B[a]P) is of particular concern because of its carcinogenic, mutagenic, and teratogenic properties (3–5). Generated mainly through incomplete combustion and released from industrial, petroleum, vehicular, and urban sources, B[a]P is widely detected in aquatic environments (6–10). Facilitates accumulation in fish through waterborne and dietary exposure, potentially causing significant physiological and pathological disturbances (11–13).

The toxicity of B[a]P is primarily associated with its metabolic activation by cytochrome P450 enzymes, particularly CYP1A, which leads to the formation of reactive metabolites and excessive generation of reactive oxygen species (ROS) (14, 15). The resulting oxidative stress disrupts cellular homeostasis through lipid peroxidation, protein oxidation, DNA damage, and mitochondrial dysfunction, ultimately impairing normal cellular functions (16–18). Consequently, B[a]P exposure has been associated with numerous adverse effects in fish, including oxidative stress, immunotoxicity, developmental abnormalities, behavioral disturbances, neurotoxicity, cardiotoxicity, and organ-specific tissue damage (17, 19, 20).

B[a]P exposure can adversely affect multiple physiological systems in fish, particularly the immune and nervous systems. Its immunotoxic effects include impaired immune responses, altered phagocytic activity, apoptosis, and dysregulation of immune-related genes, largely associated with ROS production and oxidative stress (21–24). Similarly, B[a]P-induced oxidative stress and accumulation of its metabolites in nervous tissues can disrupt neurotransmission, cause neuronal damage, and lead to behavioral and neurodevelopmental abnormalities (10, 25–27). B[a]P may also impair cardiovascular development and function, particularly during early life stages when the developing heart is highly susceptible to toxic stress (10, 28–30).

Nitric oxide (NO), myeloperoxidase (MPO), and immunoglobulin M (IgM) are important biomarkers of fish immune function. Nitric oxide produced by activated macrophages contributes to pathogen elimination and immune regulation, whereas MPO reflects neutrophil activation and inflammatory responses (28, 31, 32). In addition, IgM is the primary antibody in teleost fish and plays a key role in humoral immunity (33, 34). Acetylcholinesterase (AChE) is a sensitive biomarker of neurotoxicity, as alterations in its activity reflect disturbances in neurotransmission and neuronal function (35, 36). Histopathological studies have reported tissue degeneration, necrosis, edema, hemorrhage, and inflammatory infiltration following PAH exposure, highlighting the sensitivity of cardiac tissues to pollutant-induced damage (13, 37). Furthermore, histochemical techniques such as periodic acid–Schiff (PAS) and toluidine blue staining can reveal changes in tissue glycogen content and cellular organization that may not be detected by routine histology alone (36, 38). Therefore, the combined assessment of immune, AChE activity, histopathology, and histochemistry provides a comprehensive approach for evaluating the immunotoxic, neurotoxic, and cardiotoxic effects of B[a]P in fish.

The grass carp, *Ctenopharyngodon idella*, is one of the most important freshwater fish species worldwide and represents a cornerstone of global aquaculture production (39). Owing to its rapid growth rate, high feed conversion efficiency, herbivorous feeding habits, and economic value, grass carp contributes substantially to freshwater fish production in many countries, particularly throughout Asia and increasingly in other regions of the world (40). Beyond its commercial significance, grass carp plays an important ecological role in aquatic ecosystems through the regulation of aquatic vegetation and nutrient cycling. Consequently, maintaining the health and sustainability of grass carp populations is of considerable ecological and economic importance (39, 40).

Knowledge of B[a]P-induced cardiotoxicity in freshwater fish remains limited, particularly regarding cardiac histopathological and histochemical responses in juvenile grass carp (*Ctenopharyngodon idella*) (29, 41). Moreover, integrated assessments of the immunotoxic, neurotoxic, and cardiotoxic effects of B[a]P in this species are scarce. Therefore, this study investigated the effects of waterborne B[a]P exposure on immune function, neurotoxicity, and cardiotoxicity in juvenile *C. idella*. Immune responses were assessed using MPO, NO, NBT, and IgM, while neurotoxicity and cardiotoxicity were evaluated through AChE activity and histopathological/histochemical analyses of brain and cardiac tissues, respectively. We hypothesized that increasing B[a]P concentrations would cause dose-dependent alterations in immune function, neural integrity, and cardiac structure. This integrated approach provides further insight into B[a]P-induced multi-organ toxicity and its potential ecological implications in freshwater fish.

## 2 Materials and methods

### 2.1 Ethical statement

All experimental procedures involving grass carp (*Ctenopharyngodon idella*) larvae were conducted in accordance with internationally accepted guidelines for the care and use of laboratory animals and the ARRIVE guidelines. The study was reviewed and approved by the Ethics Committee of the Faculty of Science, Al-Azhar University (Assiut Branch), Egypt, under ethical approval No. AZHAR 12-2025.

### 2.2 Chemicals

Benzo[a]pyrene (B[a]P; ≥96% purity) was obtained from Sigma-Aldrich (St. Louis, MO, USA, CAS no:50-32-8). A stock solution of B[a]P was prepared by dissolving the compound in dimethyl sulfoxide (DMSO; analytical grade, Sigma-Aldrich, CAS no: 67-68-5) and stored in the dark at 4°C to prevent degradation. The final concentration of DMSO in exposure tanks did not exceed 0.01%, a level considered non-toxic to fish (42).

### 2.3 Fish acclimatization

One hundred and fifty juvenile grass carp (*Ctenopharyngodon idella*) (approximately one month old; average body weight: 5 ± 1 g) were obtained from a local aquaculture farm verified to be disease-free. Prior to the experimental exposure, larvae underwent a 14-day acclimation period under controlled laboratory conditions. Fish were maintained in aerated, dechlorinated freshwater tanks (capacity: 100 L) at pH (7.23 ± 0.27), temperature (24.8 ± 1.2°C), salinity (0.31 ± 0.05 ppt), light/dark cycle (12:12 h), and ammonia concentration (0.26 ± 0.05 mg/L) were monitored daily using a HORIBA multiparameter water quality meter from Japan. Dissolved oxygen levels were maintained above 80% saturation through continuous aeration. The experimental procedures followed the recommendations outlined in OECD Test Guideline No. 203, Fish, Acute Toxicity Testing (OECD, 2019), with appropriate modifications according to the objectives and experimental conditions of the present study. During acclimation, fish were fed a commercial pelleted diet (SKRETTING, Egypt) twice daily consisting of 30% crude protein, 6% crude lipid, 10% crude fiber, 12% moisture, and 5% ash, supplemented with vitamins and minerals. Water quality parameters (ammonia, nitrite, nitrate) were monitored daily and maintained within optimal ranges for larval development.

### 2.4 Experimental design and sampling

Following acclimation, grass carp juvenile were randomly divided into four groups (n=18 per group, distributed across 3 tanks per group with 6 fish per 10-L tank): a control group (water with DMSO concentration at 0.01%) and three treatment groups exposed to B[a]P at concentrations of 1, 10, and 100 µmol/L (25, 30, 43). The exposure concentrations reported in this study represent nominal concentrations. These concentrations were chosen to evaluate concentration-related biological responses across a broad exposure range and to facilitate comparison with previously published toxicological data. The selected concentrations were not intended to represent typical environmental levels of B[a]P but rather to characterize its toxicological potential under controlled laboratory conditions. Although fresh B[a]P solutions were prepared daily and a semi-static exposure system with 50% daily water renewal was employed to minimize concentration loss, the actual dissolved B[a]P concentrations were not analytically verified. Therefore, potential losses resulting from adsorption to tank surfaces, suspended particles, feed residues, or organic matter cannot be excluded (44, 45). Fish were exposed to their respective treatments for a period of 14 days under semi-static conditions. During the exposure period, fish were fed twice daily with a commercial diet, and uneaten food and waste were removed promptly to maintain water quality (46). The selected exposure concentrations of B[a]P (1, 10, and 100 µmol/L) were based on previous toxicological studies that have successfully demonstrated dose-dependent physiological, biochemical, and genotoxic responses in fish models (25, 30, 43).

Fish were fasted for 12 h before sample collection to minimize the potential influence of feeding on immune and biochemical parameters. At the end of the 14-day exposure period, fish were anesthetized and sampled for the subsequent biochemical, immunological, and histopathological analyses. Blood samples were collected from the caudal vein. The collected blood was allowed to clot at room temperature for 30 min and then centrifuged at 3,000 × g for 10 min at 4°C. The resulting serum was carefully separated, transferred into sterile microcentrifuge tubes, and stored at −80°C until biochemical and immunological analyses were performed.

### 2.5 Immune and neurotoxicity biomarkers

Serum myeloperoxidase (MPO) activity was determined according to the method described by (47). Briefly, 30 μL of serum was diluted with 370 μL of Hank’s Balanced Salt Solution (HBSS) without Ca^2+^ and Mg^2+^. Subsequently, 100 μL of 3,3′,5,5′-tetramethylbenzidine dihydrochloride (TMB; 0.1 mg mL^−1^) and 100 μL of freshly prepared 0.006% hydrogen peroxide (H_2_O_2_) were added to initiate the reaction. The change in absorbance was monitored kinetically at 450 nm using a microplate reader. Nitric oxide (NO) levels were determined according to the method of (48). Briefly, nitric oxide production was estimated indirectly by measuring nitrite (NO_2_^1^), a stable product of NO metabolism. Appropriate volumes of serum samples were mixed with Griess reagent, and the resulting chromophore was measured spectrophotometrically at 540–550 nm. Nitrite concentrations were calculated using a sodium nitrite standard curve and expressed as μmol L^−1^. Serum immunoglobulin M (IgM) levels were quantified using a commercial enzyme-linked immunosorbent assay (ELISA) kit (Catalog No. CSB-E12045Fh, Cusabio Biotech Co., Ltd.) according to the manufacturer’s instructions. Briefly, serum samples and standards were added to microplate wells pre-coated with antibodies specific for IgM and incubated under the recommended conditions. After washing, enzyme-conjugated detection antibodies were added, followed by substrate solution. The reaction was terminated with stop solution, and absorbance was measured at 450 nm using a microplate reader. IgM concentrations were calculated from a standard curve and expressed as μg mL^−1^. The nitro blue tetrazolium test (NBT) assay was conducted based on the technique of (49). Briefly, equal volumes of heparinized blood and 0.2% NBT solution were mixed and incubated at room temperature for 30 min. During incubation, reactive oxygen species generated by activated phagocytic cells reduced the yellow NBT dye to blue formazan deposits. After incubation, the reaction product was solubilized, and absorbance was measured spectrophotometrically at 540 nm. Acetylcholinesterase (AChE) activity was determined according to the method of Ellman et al. (1961). using acetylthiocholine iodide as the substrate and 5,5′-dithiobis-(2-nitrobenzoic acid) (DTNB) as the chromogenic reagent. The reaction mixture consisted of tissue homogenate, phosphate buffer (pH 7.4), DTNB, and acetylthiocholine iodide. The increase in absorbance resulting from the formation of 5-thio-2-nitrobenzoate was measured spectrophotometrically at 412 nm. Enzyme activity was normalized to the total protein content of each sample, which was determined using the Bradford method, and expressed as units per milligram of protein (U/mg protein) (50).

### 2.6 Histopathological analysis

Brain and Heart were sampled from carps to explore histological damage (n = 6 per experimental group). Tissue samples were washed in a NaCl saline solution and then fixed using buffered formalin solution (10%) for 24 h. Succeeding fixation, the tissues were embedded in paraffin wax. Later, serial sections (5 μm thick) were obtained from the paraffin blocks, then stained using hematoxylin and eosin (H&E) for histological investigation, following the method described by (38), periodic acid–Schiff’s (PAS) reaction for the detection of carbohydrates and glycoproteins, and Toluidine blue for the visualization of acidic tissue components such as nucleic acids and mast cell granules. Stained slides were examined and photographed using an Olympus microscope (BX50F4, Olympus Optical Co. LTP, Japan) and a camera (OMAX, 14MP, China) (51). Histopathological lesions were evaluated semi-quantitatively according to their extent and severity using the following scoring system: absent (–), no detectable lesion; mild (+), lesion affecting <25% of the examined tissue; moderate (++), lesion affecting approximately 25–50% of the tissue; severe (+++), lesion affecting approximately 50–75% of the tissue; and very severe (++++), lesion affecting >75% of the examined tissue.

### 2.7 Statistical analysis

For biochemical and histopathological analyses, six fish were randomly sampled from each treatment group at the end of the exposure period. Each fish was considered a biological replicate, and each biochemical measurement was performed once for each biological sample. No technical replicates were included in the statistical analysis. Data are presented as the mean ± standard error (SE). Statistical analyses were performed using GraphPad Prism version 9.0 (GraphPad Software, San Diego, CA, USA). Data normality was assessed using the Shapiro–Wilk test, while homogeneity of variances was evaluated using Levene’s test. Differences among groups were analyzed using one-way analysis of variance (ANOVA), followed by Tukey’s multiple-comparison *post hoc* test. The significance level of p< 0.05 was considered statistically significant. Individual fish were analyzed as biological replicates. Although fish were distributed among three replicate tanks per treatment, tank was not incorporated as a factor in the statistical analysis.

## 3 Results

### 3.1 Immune biomarker

**Figure 1:**
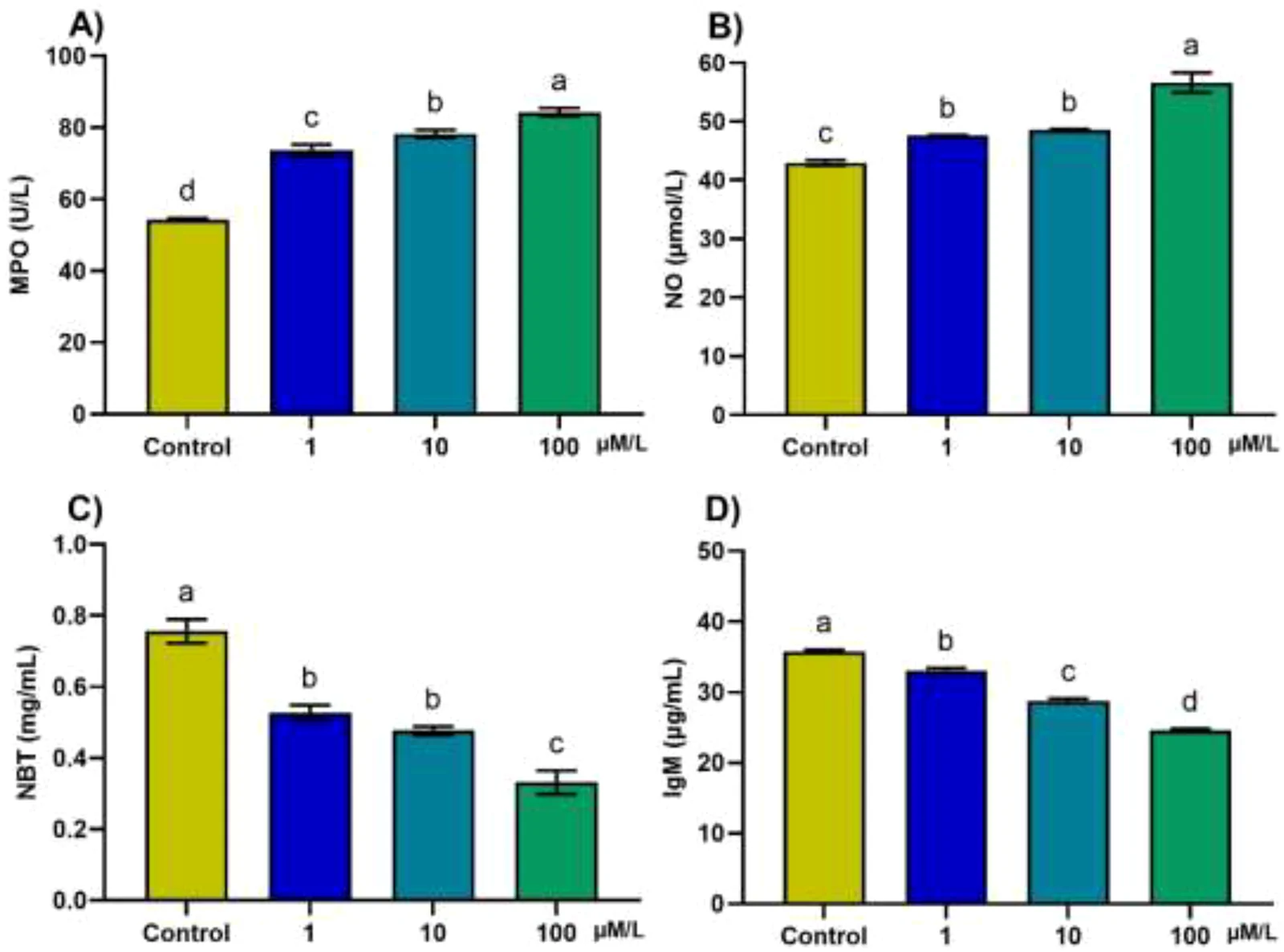
Effect of Benzo[a]pyrene (B[a]P) exposure on immune parameters in grass carp (*Ctenopharyngodon idella*) juvenile after 14 days at concentrations of 1, 10, and 100 µmol/L. The measured parameters include: **(A)** Myeloperoxidase (MPO) Activity, **(B)** Nitric oxide (NO), **(C)** Nitro blue tetrazolium (NBT), and **(D)** immunoglobulin (IgM). Data are presented as mean ± standard deviation (SD). Statistical significance was determined using one-way ANOVA followed by Tukey’s multiple-comparison *post hoc* test. Superscript letters indicate significant differences compared to the control group.

Exposure to B[a]P significantly affected immune parameters in grass carp (*Ctenopharyngodon idella*) juvenile (**Figure 1**). MPO activity increased significantly among treatments (F_3,20_ = 131.6, *p* < 0.0001), with *post hoc* analysis showing significant increase at 1, 10, and 100 µmol/L (p < 0.0001) compared with the control. Similarly, NO was significantly increased (F_3,20_ = 40.41, *p* < 0.0001), with *post hoc* comparisons revealing significant increases at 1 and 10 µmol/L (*p* < 0.01) and significant increases at 100 µmol/L (*p* < 0.0001). In contrast, NBT levels decreased significantly in fish across treatments (F_3,20_ = 44.12, *p* < 0.0001), with *post hoc* analysis indicating significant reduction at 1, 10, and 100 µmol/L (*p* < 0.0001). Likewise, IgM levels decreased significantly (F_3,20_ = 268.8, *p* < 0.0001) in fish, with *post hoc* tests confirming significant decreases at all exposure concentrations (*p* < 0.0001).

### 3.2 Neurotoxicity biomarker

**Figure 2:**
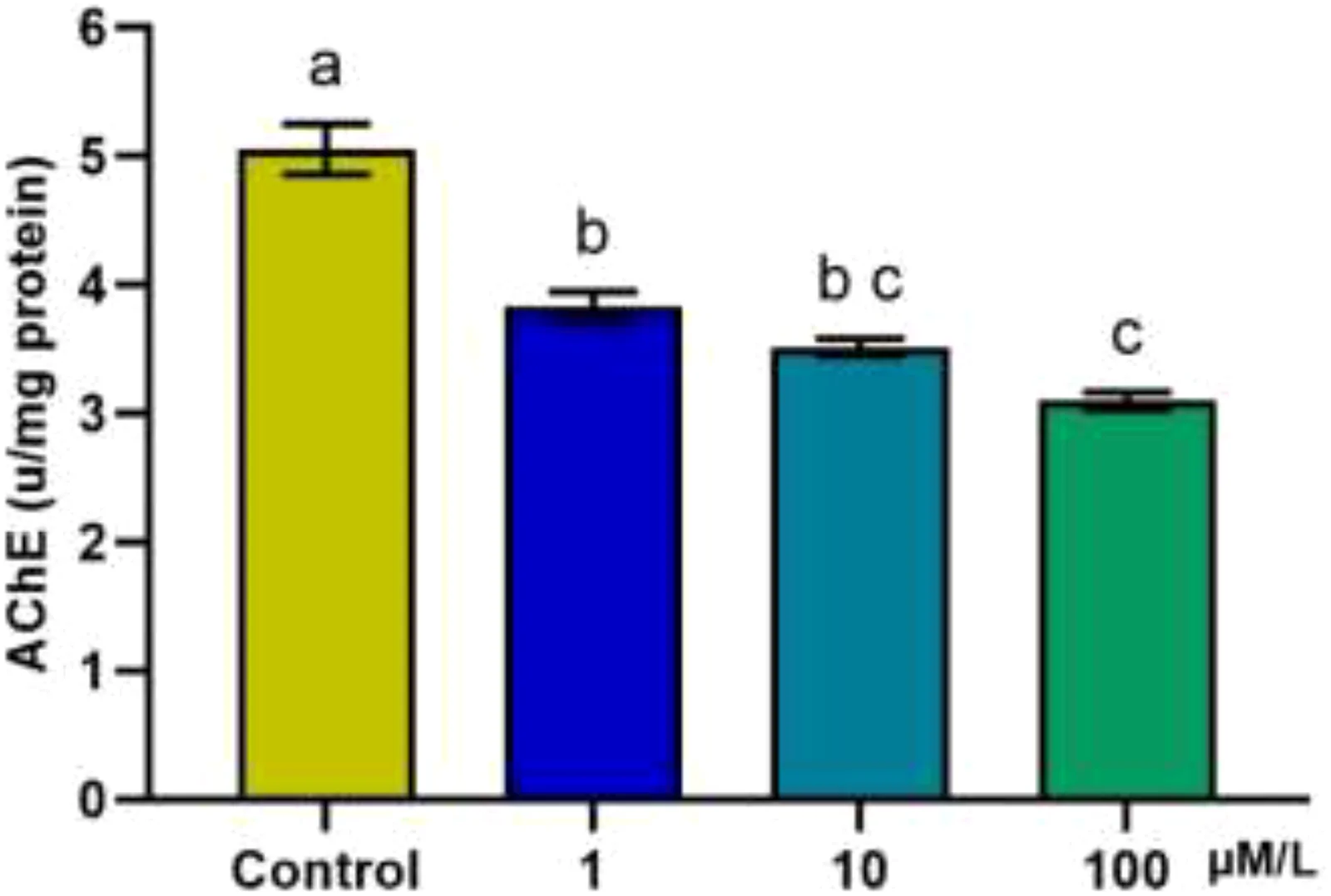
Effect of Benzo[a]pyrene (B[a]P) exposure on acetylcholinesterase (AChE; U/mg protein) activity in grass carp (*Ctenopharyngodon idella*) juvenile after 14 days at concentrations of 1, 10, and 100 µmol/L. Data are presented as mean ± standard deviation (SD). Statistical significance was determined using one-way ANOVA followed by Tukey’s multiple-comparison *post hoc* test. Superscript letters indicate significant differences compared to the control group.

Exposure to B[a]P significantly affected AChE activity in grass carp (*Ctenopharyngodon idella*) juvenile (**Figure 2**). AChE activity decreased significantly across treatments (F_3,20_ = 46.39, *p* < 0.0001) compared with the control group. *Post hoc* analysis revealed significant inhibition at 1, 10 and 100 µmol/L (*p* < 0.0001).

### 3.3 Histopathological and histochemical examination of brain tissue

**Figure 3:**
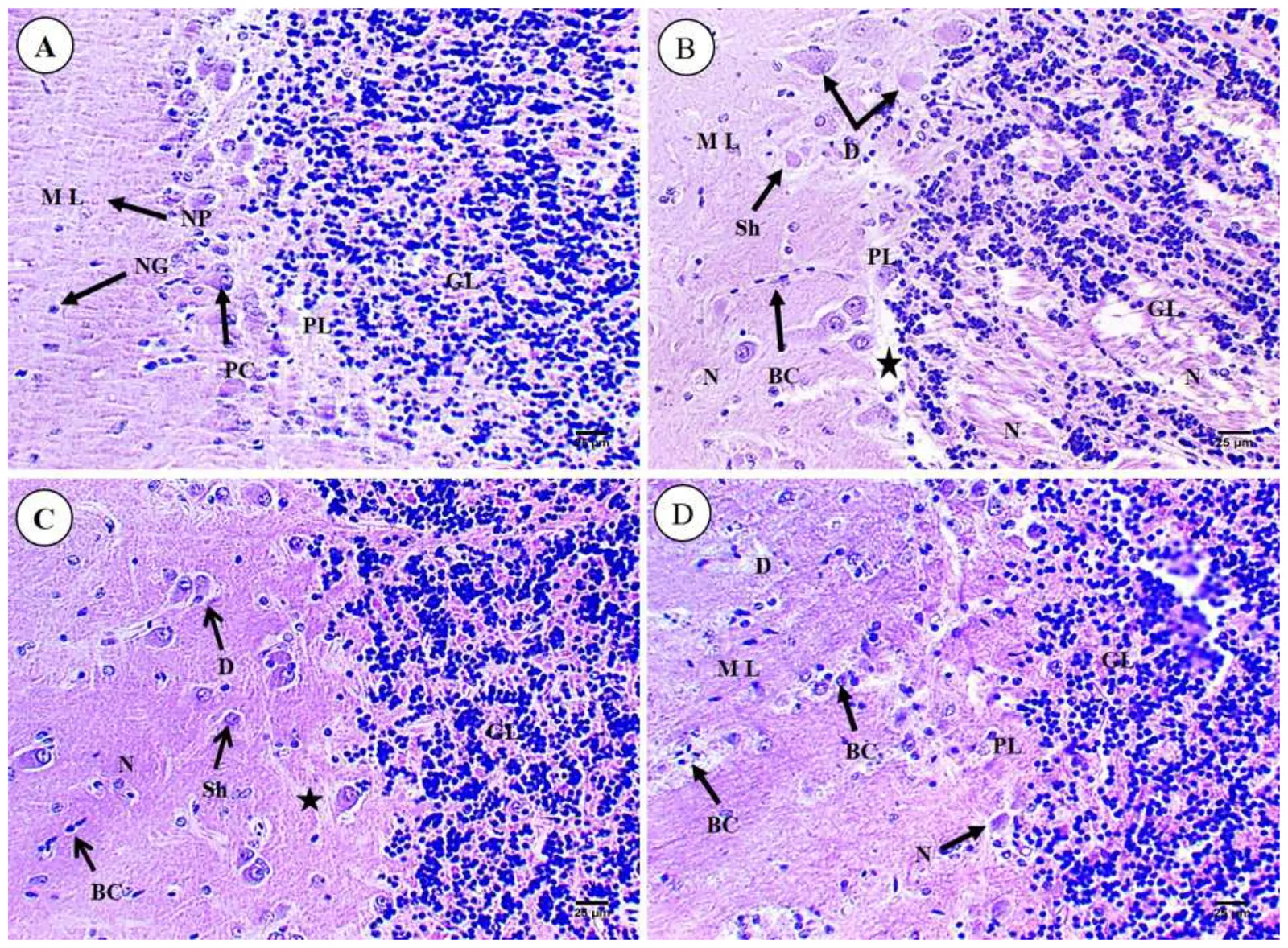
Histopathological changes in the cerebellum of juvenile grass carp (*Ctenopharyngodon idella*) following 14-day exposure to nominal concentrations of B[a]P (H&E, ×400). **(A)** Control fish, **(B)** Fish exposed to 1 µM B[a]P, **(C)** Fish exposed to 10 µM B[a]P, and **(D)** Fish exposed to 100 µM B[a]P. ML, molecular layer; PL, Purkinje cell layer; GL, granular layer; PC, Purkinje cells; NP, neuropil; NG, neuroglial cells; BC, blood capillaries; D, degeneration; N, necrosis; Sh, shrunken cells; ★, necrotic lesions.

Histological examination of haematoxylin and eosin (H&E)-stained cerebellar sections from the control group revealed the normal architecture of the cerebellum, which consisted of three distinct layers: the molecular layer, the Purkinje cell layer, and the granular layer. The molecular layer exhibited a homogeneous neuropil containing a few neuroglial cells. Purkinje cells were arranged in a single orderly row at the interface between the molecular and granular layers. These cells possessed homogeneous basophilic cytoplasm, vesicular eccentric nuclei, and centrally located nucleoli, and some exhibited prominent dendritic processes. The granular layer contained densely packed neuroglial cells with small, deeply stained nuclei distributed throughout the neuropil (**Figure 3A**).

Exposure to 1 µM B[a]P induced degenerative changes in all layers of the cerebellar cortex. The molecular layer exhibited neuropilar degeneration and necrotic areas (malacia), accompanied by disruption of the normal arrangement of Purkinje cells. Many Purkinje cells were displaced into the molecular layer, while others showed signs of degeneration, including cellular shrinkage and nuclear pyknosis. Extensive necrotic areas were observed at the interface between the molecular and granular layers. Increased vascularization and congestion of blood capillaries were also evident in the molecular layer. The granular layer showed severe neuropilar degeneration and necrosis, accompanied by a reduction in the number of neuroglial cell aggregates (**Figure 3B**).

The cerebellum of fish exposed to 10 µM B[a]P exhibited marked degeneration of the molecular layer, with extensive neuropilar degeneration and necrotic foci. The regular alignment of Purkinje cells was lost, and many cells were randomly dispersed throughout the molecular layer. Most Purkinje cells retained homogeneous basophilic cytoplasm and vesicular nuclei with prominent nucleoli, whereas others displayed degenerative changes, including shrinkage and cytoplasmic deterioration. Increased numbers of blood capillaries were observed, and focal necrotic areas were present within the Purkinje cell layer. In contrast to the 1 µM group, the granular layer showed partial recovery, characterized by a more normal distribution of neuroglial cells and a reduction in neuropilar degeneration, although vascular congestion remained evident (**Figure 3C**).

Fish exposed to 100 µM B[a]P exhibited severe pathological alterations in the cerebellar cortex. The molecular layer showed extensive degeneration and necrosis of the neuropil. The Purkinje cell layer was severely disrupted, with widespread degeneration and loss of its normal organization. Numerous Purkinje cells exhibited pyknotic nuclei and other signs of cellular degeneration. Marked angiogenesis and vascular congestion were observed within the molecular layer. In addition, many neurons displayed irregular morphologies and deformed eccentric nuclei, indicating severe neurotoxicity (**Figure 3D**).

**Figure 4:**
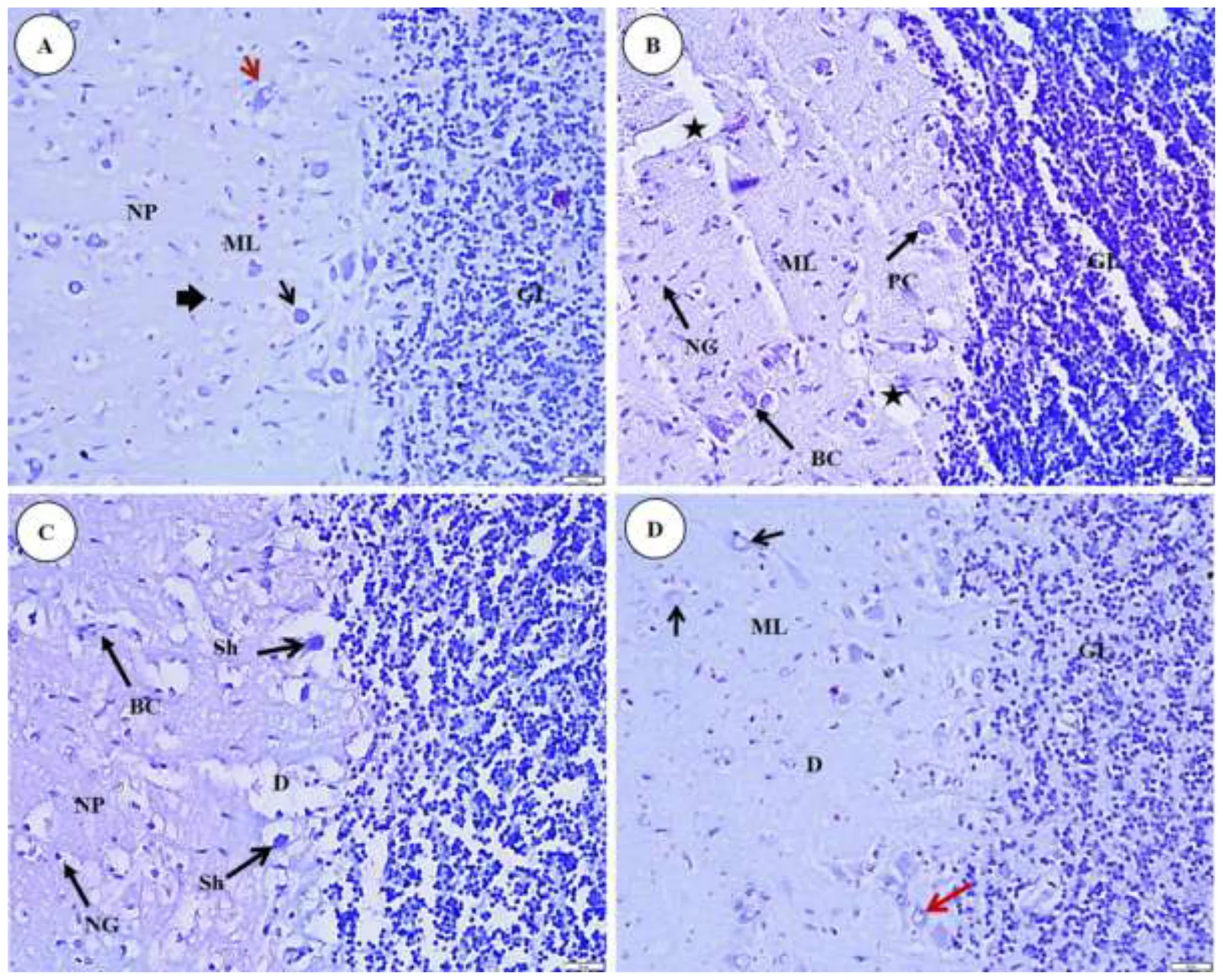
Histochemical changes in the cerebellum of juvenile grass carp (*Ctenopharyngodon idella*) following 14-day exposure to nominal concentrations of B[a]P (Toluidine blue, ×400). **(A)** Control fish, **(B)** fish exposed to 1 µM B[a]P, **(C)** fish exposed to 10 µM B[a]P, and **(D)** fish exposed to 100 µM B[a]P. ML, molecular layer; GL, granular layer; NP, neuropil; NG, neuroglial cells; BC, blood capillaries; D, degeneration; Sh, shrunken cells; ★, necrotic lesions.

Toluidine blue staining was used to evaluate Nissl substance (RNA-containing granules) within cerebellar neurons. In the control group, Purkinje cells were normally distributed at the boundary between the molecular and granular layers. Abundant Nissl granules stained intensely blue and surrounded the unstained nuclei of Purkinje cells, which appeared either rounded (black arrow) or flask-shaped (red arrow). The neuropil of both molecular and granular layers exhibited faint blue staining, while glial cells showed distinct blue coloration (**Figure 4A**). In fish exposed to 1 µM B[a]P, Purkinje cells exhibited chromatolysis, characterized by dissociation and depletion of Nissl granules with reduced staining intensity. The number of Purkinje cells decreased, and many cells migrated into the molecular layer. Increased numbers of glial cells, degeneration of the neuropil, and vascular congestion were also observed (**Figure 4B**). Exposure to 10 µM B[a]P resulted in shrunken Purkinje cells with eccentrically located nuclei surrounded by unstained pericellular spaces. Marked dissociation of Nissl granules and neuropilar degeneration were evident. Only a few Purkinje cells remained intact, while increased numbers of glial cells and congested blood capillaries were observed throughout the affected tissue (**Figure 4C**). In fish exposed to 100 µM B[a]P, clusters of Purkinje cells with eccentric nucleoli (red arrow) were observed. These cells contained sparse and weakly stained Nissl granules, indicating substantial loss of RNA-rich material. Degenerative changes and neuronal malacia were clear in many Purkinje cells (black arrow). Increased numbers of glial cells, mild neuropilar degeneration, and prominent blood capillaries were also observed (**Figure 4D**).

### 3.4 Histopathological and histochemical examination of heart tissue

The heart of grass carp consists of four chambers: the sinus venosus, atrium, ventricle, and bulbus arteriosus. Histologically, the cardiac wall is composed of three layers: the epicardium, myocardium, and endocardium. The atrium is characterized by a relatively thin wall composed mainly of the epicardium and endocardium, whereas the ventricle possesses a thicker myocardium formed by densely packed cardiomyocytes (**Table 1**).

**Table 1:** Semi-quantitative scoring of histopathological lesions in the cerebellum and ventricle of juvenile grass carp (*Ctenopharyngodon idella*) following 14-day exposure to nominal concentrations of benzo[a]pyrene (B[a]P).

Lesions	Control	1 µM	10 µM	100 µM
Brain				
Disorganization of cerebellar cortex	–	+	++	++++
Purkinje cell degeneration	–	++	+++	++++
Purkinje cell loss/disruption	–	+	+++	++++
Neuropilar necrosis	–	+	++	++++
Neuronal shrinkage	–	+	++	+++
Pyknotic nuclei	–	+	++	++++
Vacuolar degeneration	–	++	+++	++++
Vascular congestion	–	+	++	+++
Hemorrhage	–	–	+	++
Reduction of neuroglial cells	–	+	++	+++
Heart				
Epicardial degeneration	–	++	+++	++++
Subepicardial edema	–	++	+++	++++
Cardiomyocyte degeneration	–	++	+++	++++
Cardiomyocyte necrosis	–	+	+++	++++
Myocardial disorganization	–	++	+++	++++
Trabecular thickening/disorganization	–	++	+++	++++
Lacunar space alteration	–	++	+++	+++
Vascular congestion	–	+	+++	++++
Hemorrhage	–	+	++	+++
Pyknotic nuclei	–	+	++	++++

Histopathological lesions were evaluated semi-quantitatively according to their extent and severity using the following scoring system: absent (–), no detectable lesion; mild (+), lesion affecting <25% of the examined tissue; moderate (++), lesion affecting approximately 25–50% of the tissue; severe (+++), lesion affecting approximately 50–75% of the tissue; and very severe (++++), lesion affecting >75% of the examined tissue.

#### 3.4.1 Histopathological and histochemical examination of the atrium

**Figure 5:**
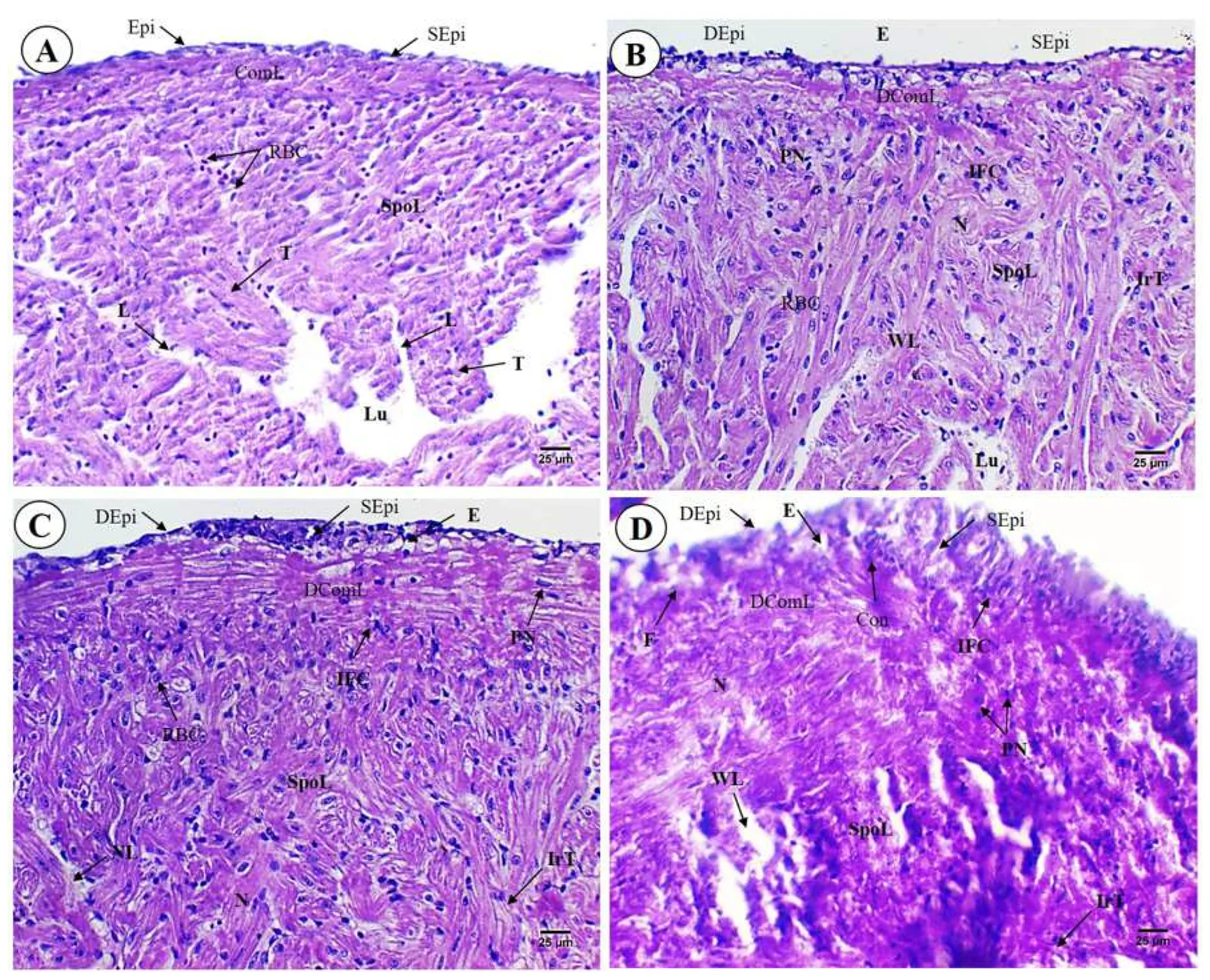
Histopathological changes in the atrium of juvenile grass carp (*Ctenopharyngodon idella*) following 14-day exposure to nominal concentrations of B[a]P (H&E, ×400). **(A)** Control fish, **(B)** fish exposed to 1 µM B[a]P, **(C)** fish exposed to 10 µM B[a]P, and **(D)** fish exposed to 100 µM B[a]P. Epi, epicardium; ComL, compact myocardial layer; SpoL, spongy layer; T, trabeculae; L, lacunae; Lu, atrial lumen; RBC, erythrocytes; DEpi, epicardial degeneration; SEpi, subepicardial layer; E, edema; DComL, myocardial degeneration; IFC, inflammatory cell infiltration; PN, pyknotic nuclei; N, necrosis; IrT, irregular trabeculae; WL, widened lacunae; F, fat droplets; Con, congestion.

The atrial tissue of the control group exhibited a delicate structure that was more finely trabeculated than that of the ventricle. The outer layer (epicardium) consisted of a simple squamous epithelium supported by a narrow subepicardial layer of loose connective tissue. The myocardium formed a compact layer composed of regularly arranged circular cardiomyocytes with deeply stained nuclei. Small spaces containing erythrocytes were observed between the myocardial fibres. The inner endocardial (spongy) layer consisted of thin, irregular trabeculae with cardiomyocytes arranged in different orientations. These cells exhibited acidophilic cytoplasm and spindle-shaped nuclei. Narrow lacunae containing erythrocytes were observed between the trabeculae (**Figure 5A**). Exposure to 1 µM B[a]P induced degeneration of the epicardial epithelium and edema within the subepicardial connective tissue. The compact myocardial layer became thinner and more fragile, with evident degeneration of cardiomyocytes. The apical region of the spongy endocardial layer showed infiltration of mononuclear inflammatory cells and pyknotic nuclei of degenerated cardiomyocytes. The trabeculae appeared irregular, separated, and variably oriented. Cardiomyocytes exhibited acidophilic cytoplasm and vesicular nuclei. Widened lacunae containing erythrocytes were observed between the trabeculae (**Figure 5B**). In fish exposed to 10 µM B[a]P, the epicardium exhibited marked irregularity and degeneration. The subepicardial layer was enlarged and contained edematous vacuoles together with focal accumulations of mononuclear inflammatory cells. The compact myocardial layer was enlarged and showed degeneration and necrosis. Numerous cardiomyocytes exhibited pyknotic nuclei, accompanied by intercellular edema. The spongy layer displayed enlarged trabeculae with irregular orientations, containing acidophilic sarcoplasm and both vesicular and pyknotic nuclei. The lacunae between trabeculae became narrower and contained erythrocytes. Focal necrotic areas were also observed (**Figure 5C**). The atria of fish exposed to 100 µM B[a]P showed severe disruption of normal tissue architecture. Degeneration and partial disintegration of the epicardium were evident, together with marked enlargement of the subepicardial layer containing numerous oedematous vacuoles. Congested blood capillaries, mononuclear inflammatory cell infiltration, and occasional lipid droplets were observed. The compact myocardial layer was widened and exhibited extensive cardiomyocyte degeneration and necrosis characterized by pyknotic nuclei. Increased intercellular edema and erythrocyte infiltration were evident. The spongy layer showed enlarged lacunae and complete fusion of trabeculae with numerous pyknotic nuclei (**Figure 5D**).

**Figure 6:**
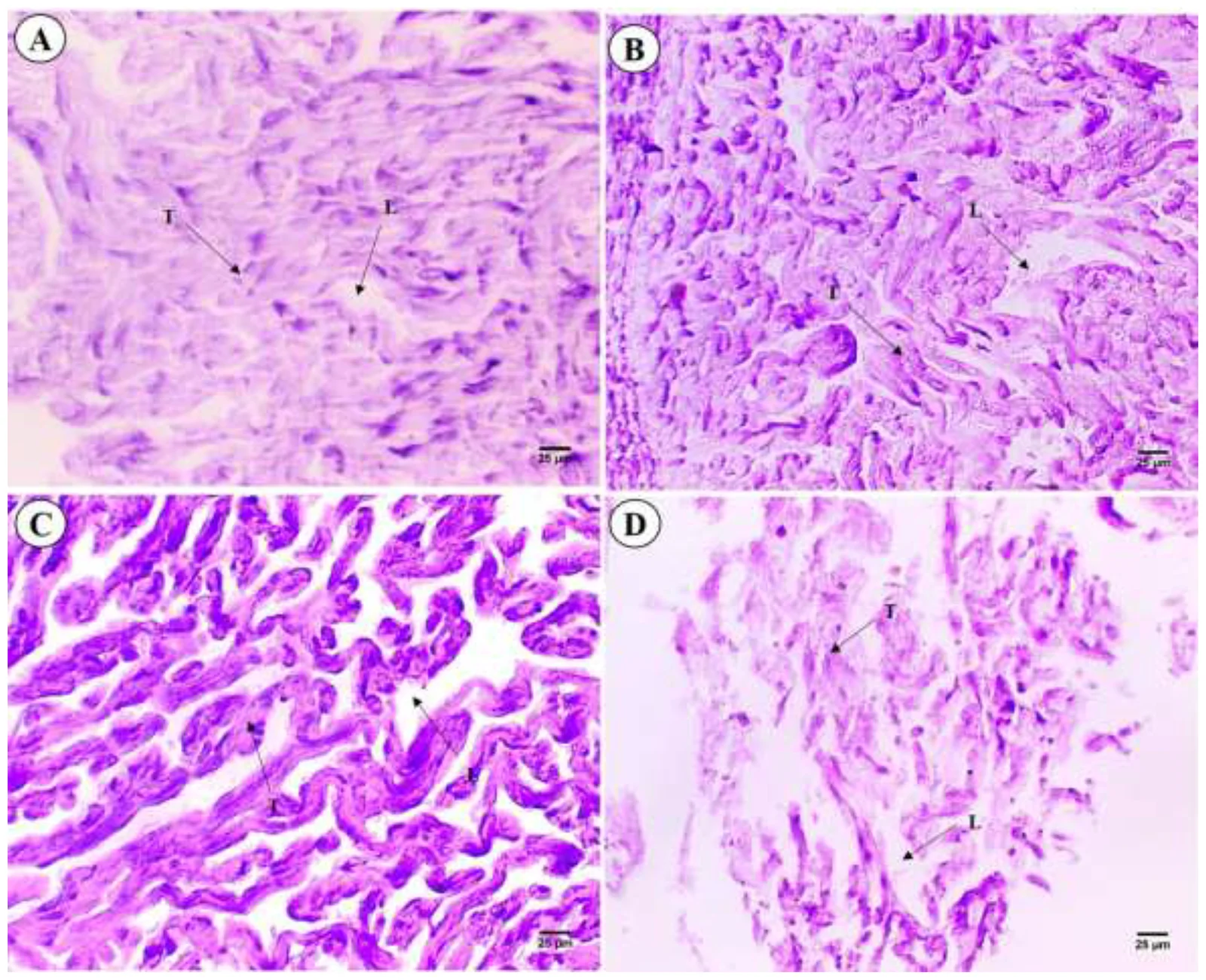
Histochemical alterations in the atrium of juvenile grass carp (*Ctenopharyngodon idella*) following 14-day exposure to nominal concentrations of B[a]P (PAS, ×400). **(A)** Control fish, **(B)** fish exposed to 1 µM B[a]P, **(C)** fish exposed to 10 µM B[a]P, and **(D)** fish exposed to 100 µM B[a]P. T, trabeculae; PAS, periodic acid–Schiff.

Periodic acid–Schiff (PAS) staining of atrial sections from the control group demonstrated a normal distribution of polysaccharides within the atrial tissue (**Figure 6A**). In fish exposed to 1 µM B[a]P, PAS-positive material increased, with fine glycogen granules and peripheral glycogen accumulation observed along the trabeculae (**Figure 6B**). Exposure to 10 µM B[a]P resulted in a further increase in polysaccharide deposition compared with both the control and low-dose groups (**Figure 6C**). In contrast, fish exposed to 100 µM B[a]P exhibited a marked reduction in polysaccharide content within the atrial tissue compared with the other experimental groups (**Figure 6D**).

**Figure 7:**
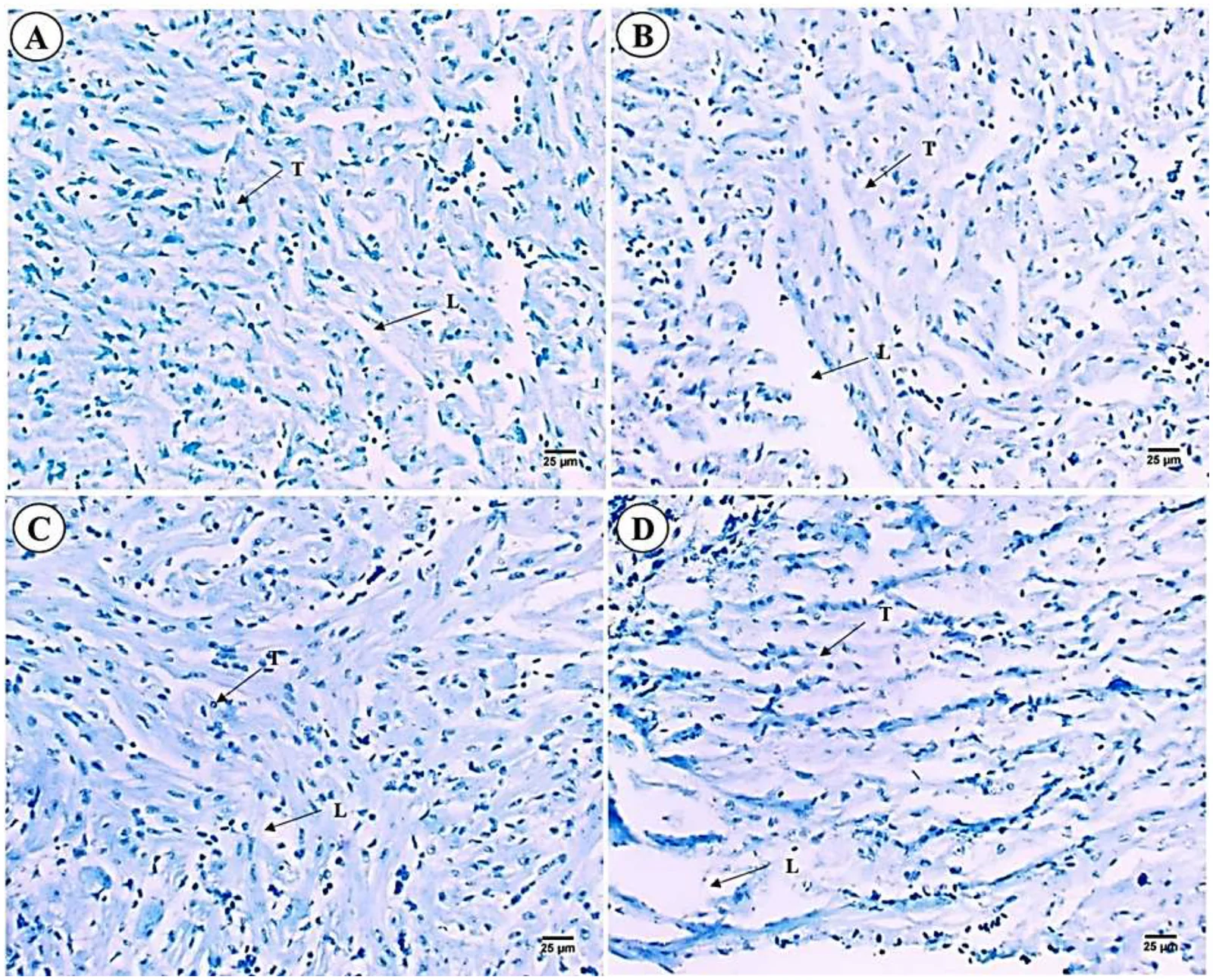
Histochemical changes in the atrium of juvenile grass carp (*Ctenopharyngodon idella*) following 14-day exposure to nominal concentrations of B[a]P (Toluidine blue, ×400). **(A)** Control fish, **(B)** fish exposed to 1 µM B[a]P, **(C)** fish exposed to 10 µM B[a]P, and **(D)** fish exposed to 100 µM B[a]P. T, trabeculae; L, lacunar spaces.

Toluidine blue staining was used to identify acidic tissue components, including carboxylates, sulfates, and phosphate radicals. In the control group, atrial tissues exhibited normal orthochromatic blue staining of the sarcoplasm and nuclei. Fish exposed to 1 µM B[a]P showed increased orthochromatic staining intensity, accompanied by an increased number of blood cell nuclei within the lacunar spaces. In the 10 µM B[a]P group, strong orthochromatic staining was observed in the trabeculae, and numerous blood cell nuclei were present within the narrowed lacunae. In contrast, atrial tissues from fish exposed to 100 µM B[a]P exhibited weaker orthochromatic staining, although blood cell nuclei remained visible within the lacunar spaces (**Figures 7A–D**).

#### 3.4.2 Histopathological and histochemical examination of the ventricle

**Figure 8:**
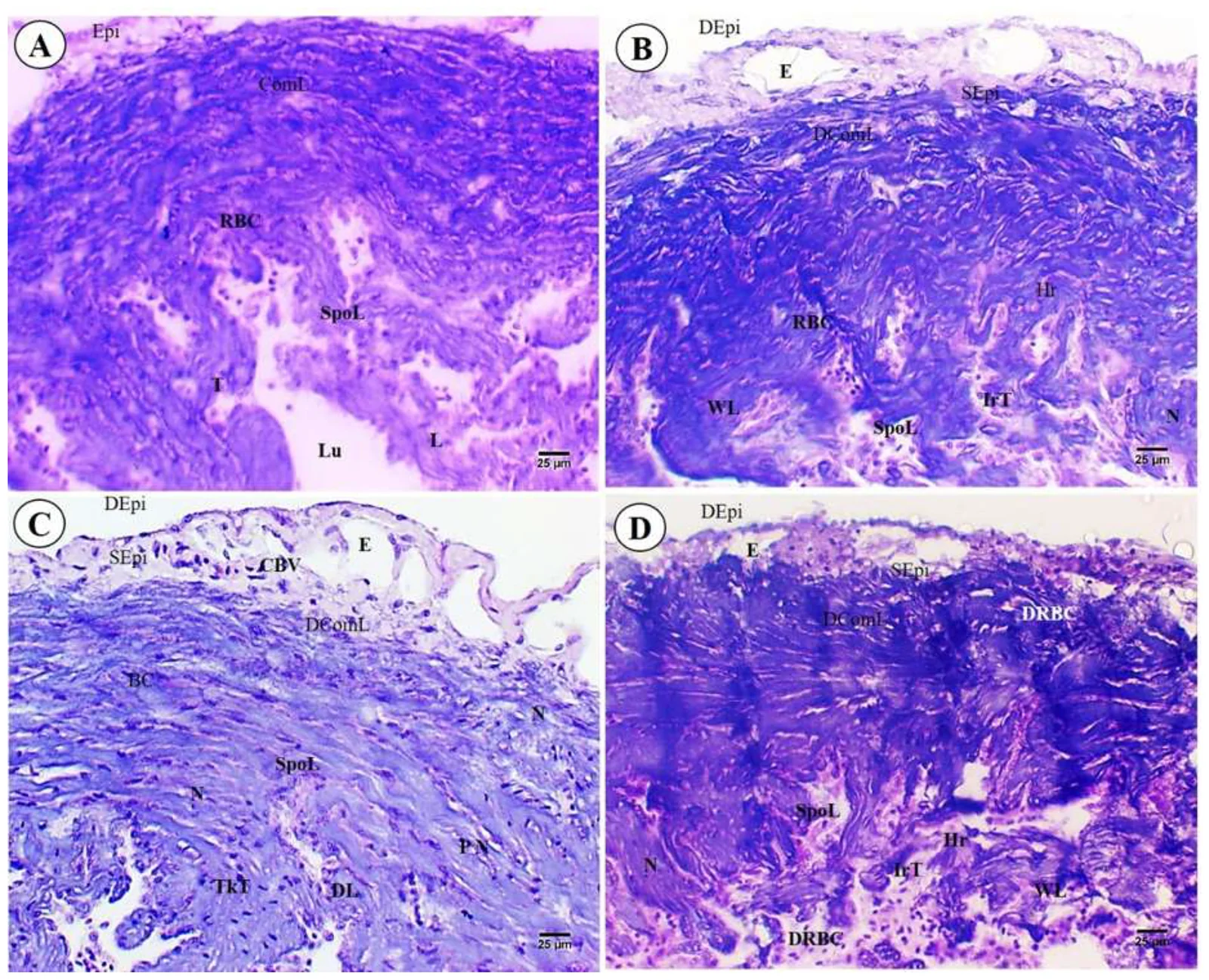
Histopathological changes in the ventricle of juvenile grass carp (*Ctenopharyngodon idella*) following 14-day exposure to nominal concentrations of B[a]P (H&E, ×400). **(A)** Control fish, **(B)** fish exposed to 1 µM B[a]P, **(C)** fish exposed to 10 µM B[a]P, and **(D)** fish exposed to 100 µM B[a]P. Epi, epicardium; ComL, compact myocardial layer; SpoL, spongy layer; T, trabeculae; L, lacunae; RBC, erythrocytes; DEpi, epicardial degeneration; SEpi, subepicardial layer; E, edema; DComL, myocardial degeneration; IrT, irregular trabeculae; WL, widened lacunae; Hr, hemorrhage; CBV, congested blood vessels; N, necrosis; TkT, thickened trabeculae; DL, reduced lacunar spaces; PN, pyknotic nuclei; DRBC, degenerated erythrocytes.

Histological examination of sagittal sections of the ventricular wall from the control group revealed the normal architecture of the heart ventricle, which consisted of three distinct layers: the epicardium, myocardium, and endocardium. The outer epicardial layer was thin and intact. The myocardium formed a compact muscular layer composed of regularly arranged circular cardiomyocytes. The inner endocardial (spongy) layer consisted of thick trabeculae, with cardiomyocytes arranged in longitudinal, circumferential, and oblique orientations. Narrow lacunar spaces containing erythrocytes, leukocytes, and macrophages were present between the trabeculae, while the ventricular lumen occupied the central region of the ventricle (**Figure 8A**). In fish exposed to 1 µM B[a]P, degeneration of the epicardial epithelium and enlargement of the subepicardial layer were observed. The latter contained loose connective tissue and numerous edematous vacuoles. The compact myocardial layer exhibited hypertrophy, with irregularly arranged cardiomyocytes showing intense hematoxylin staining and increased intercellular edema. The spongy layer contained thicker trabeculae than those of the control group, accompanied by enlarged lacunar spaces filled with various blood cells. Signs of hyperemia and hemorrhage were also evident (**Figure 8B**). Exposure to 10 µM B[a]P resulted in marked degeneration of the epicardium, together with enlargement of the subepicardial region containing congested blood vessels and extensive edema. The compact myocardial layer exhibited severe degenerative changes, including loss of normal cellular architecture and necrosis. Numerous blood capillaries engorged with erythrocytes were present between myocardial fibers. The spongy layer showed extensive necrotic areas, thickened trabeculae, and reduced lacunar spaces. Many cardiomyocytes exhibited pyknotic nuclei, indicating advanced cellular degeneration (**Figure 8C**). The ventricular tissue of fish exposed to 100 µM B[a]P displayed severe histopathological alterations. The epicardial epithelium was irregular and extensively degenerated. Although the subepicardial layer was slightly reduced compared with the 10 µM group, it still exhibited edema and contained degenerated erythrocytes with disrupted cellular boundaries and nuclear deformation. The compact myocardial layer showed extensive necrosis, loss of recognizable cardiomyocyte morphology, and increased vascularization. The spongy layer was markedly disorganized, with enlarged trabeculae lacking normal structural features, extensive necrotic regions, and widened lacunar spaces containing degenerated erythrocytes (**Figure 8D**).

**Figure 9:**
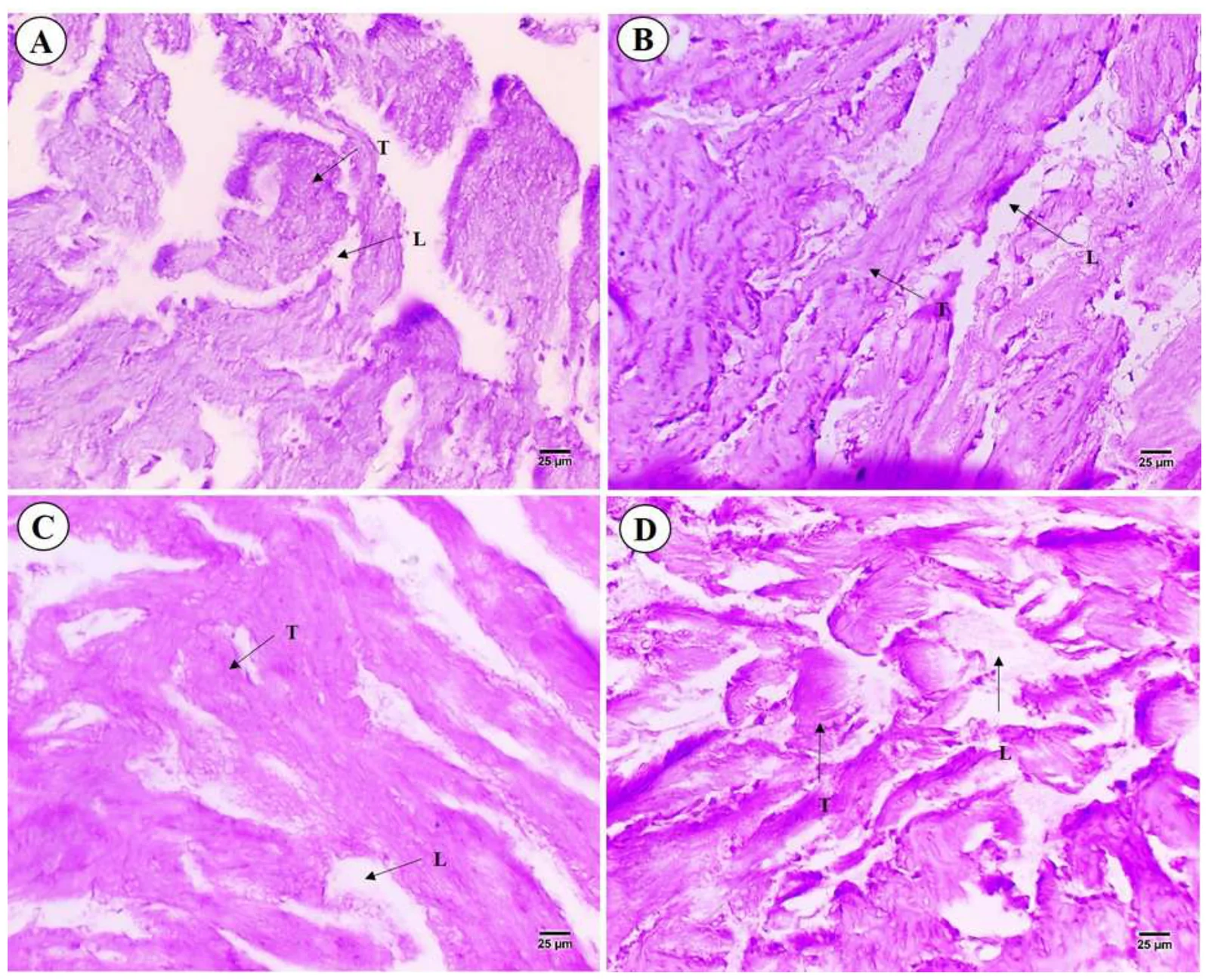
Histochemical changes in the ventricle of juvenile grass carp (*Ctenopharyngodon idella*) following 14-day exposure to nominal concentrations of B[a]P (PAS, ×400). **(A)** Control fish, **(B)** fish exposed to 1 µM B[a]P, **(C)** fish exposed to 10 µM B[a]P, and **(D)** fish exposed to 100 µM B[a]P. T, trabeculae; PAS, periodic acid–Schiff.

Periodic acid–Schiff (PAS) staining demonstrated a normal distribution of polysaccharides within the ventricular tissue of the control group (**Figure 9A**). Fish exposed to 1 µM B[a]P exhibited a heterogeneous distribution and a slight increase in polysaccharide deposition within the ventricular trabeculae (**Figure 9B**). In contrast, exposure to 10 µM B[a]P resulted in a marked reduction and dissociation of PAS-positive materials, indicating depletion of polysaccharide content compared with both the control and 1 µM groups (**Figure 9C**). However, fish exposed to 100 µM B[a]P showed a pronounced increase in polysaccharide deposition relative to the 10 µM group (**Figure 9D**).

**Figure 10:**
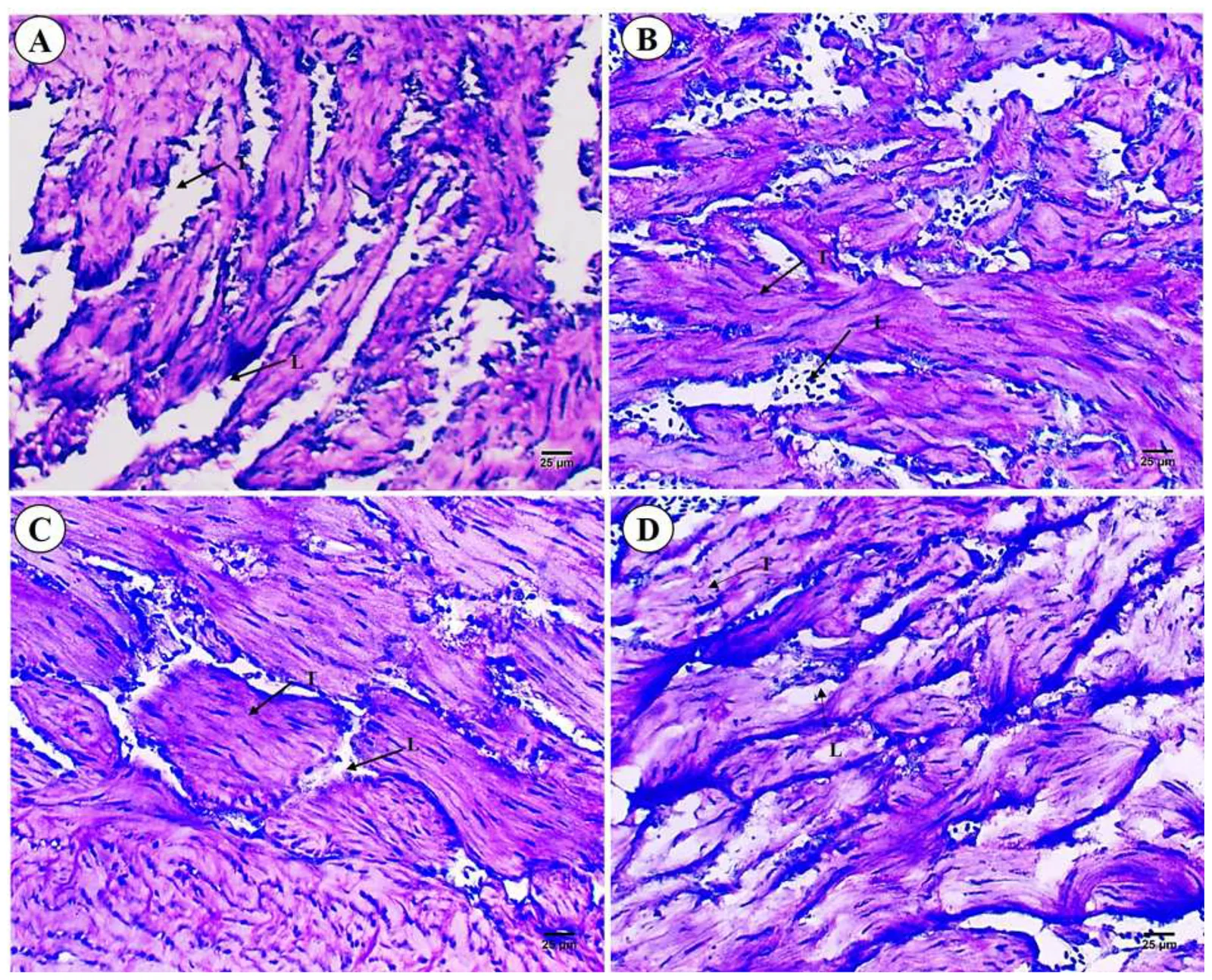
Histochemical changes in the ventricle of juvenile grass carp (*Ctenopharyngodon idella*) following 14-day exposure to nominal concentrations of B[a]P (Toluidine blue, ×400). **(A)** Control fish, **(B)** fish exposed to 1 µM B[a]P, **(C)** fish exposed to 10 µM B[a]P, and **(D)** fish exposed to 100 µM B[a]P. T, trabeculae; L, lacunar spaces.

Toluidine blue staining of ventricular tissue in the control group revealed normal metachromatic violet staining of the trabecular sheets, while the nuclei stained blue (**Figure 10A**). Fish exposed to 1 µM B[a]P exhibited increased metachromatic staining intensity within the ventricular trabeculae, accompanied by an increased number of blood cell nuclei within the lacunar spaces (**Figure 10B**). In the 10 µM B[a]P group, the ventricular tissue showed a further increase in staining intensity, particularly in the flattened and widened trabeculae, with stronger staining observed at their margins (**Figure 10C**). In contrast, fish exposed to 100 µM B[a]P exhibited a slight reduction in staining intensity, especially along the boundaries of the widened trabeculae. The lacunar spaces appeared smaller than those observed in the other treated groups (**Figure 10D**).

## 4 Discussion

The present study demonstrated that short-term exposure to benzo[a]pyrene (B[a]P) induced significant immunotoxic, neurotoxic, cardiotoxic, histopathological, and histochemical alterations in grass carp (*Ctenopharyngodon idella*) juvenile. The observed changes in immune biomarkers, including MPO, NO, NBT, and IgM, together with the inhibition of AChE activity and severe structural damage in brain and cardiac tissues, collectively indicate that B[a]P exerts pronounced multi-organ toxicity during early developmental stages. These findings are consistent with previous studies demonstrating that B[a]P and other PAHs adversely affect fish physiology through oxidative stress, inflammation, cellular degeneration, and disruption of normal tissue function (21, 22, 26).

The immune system is particularly susceptible to environmental contaminants during early developmental stages, when defence mechanisms are not yet fully developed (17, 52). The alterations indicate activation of inflammatory responses accompanied by suppression of both innate and adaptive immune functions (53–55).

The elevated MPO activity observed in B[a]P-exposed juvenile suggests enhanced neutrophil activation and inflammatory responses (56, 57). MPO is an important enzyme released by activated phagocytic cells and contributes to microbial killing through the production of highly reactive oxidants. Increased MPO activity has been widely recognized as a biomarker of inflammatory stress and pollutant-induced immune dysfunction in fish (31, 58). Likewise, the significant increase in NO levels following B[a]P exposure may reflect an enhanced inflammatory response and altered immune signalling. Because NO levels increased across the tested concentrations, this response should not be interpreted as evidence of impaired immune function at the highest concentration. Instead, the observed elevation in NO may indicate concentration-dependent modulation of inflammatory and immune processes following B[a]P exposure.

In contrast, the significant reduction in NBT activity indicates suppression of the respiratory burst response and decreased production of reactive oxygen intermediates by phagocytic cells. Similar reductions in respiratory burst activity have been reported in fish exposed to PAHs and other environmental contaminants, leading to impaired pathogen elimination and weakened immune competence (59, 60). Furthermore, the marked decrease in IgM levels provides evidence of impaired adaptive immune function. As the principal immunoglobulin in teleost fish, IgM plays a vital role in humoral immunity and protection against infectious agents. Reduced IgM concentrations have been associated with diminished disease resistance and increased susceptibility to infections following exposure to toxic pollutants (61, 62).

The observed immunotoxic, neurotoxic, and cardiotoxic responses may be associated with oxidative stress, as suggested by previous studies demonstrating that B[a]P can induce excessive reactive oxygen species production and disrupt cellular redox homeostasis in fish (17, 23, 24, 63). However, because oxidative stress biomarkers and related molecular pathways were not directly evaluated in the present study, the involvement of oxidative stress should be considered a plausible explanation rather than a mechanism confirmed by our experimental data.

Similar immunosuppressive effects of B[a]P have been reported in several aquatic species, including Japanese medaka, Nile tilapia, common carp, and mud crab, where exposure resulted in altered immune gene expression, impaired immune competence, and increased susceptibility to disease (21–23, 64).

Although AChE inhibition is commonly associated with organophosphate pesticides, accumulating evidence indicates that PAHs can also interfere with cholinergic neurotransmission through oxidative stress-mediated mechanisms (35). Excessive ROS production may damage neuronal membranes, alter enzyme conformation, and impair neurotransmitter metabolism, ultimately leading to reduced AChE activity. Similar decreases in AChE activity have been reported in fish exposed to B[a]P and other organic pollutants and were associated with behavioral abnormalities, impaired locomotor activity, and neurological dysfunction (25, 26).

Fish exposed to B[a]P showed extensive degeneration of all cerebellar layers, neuropilar necrosis, disruption of Purkinje cell organization, neuronal shrinkage, vascular congestion, and a reduction in neuroglial cell populations. The severity of these lesions generally increased with increasing B[a]P concentration, indicating a dose-dependent neurotoxic effect.

These findings are consistent with previous studies reporting severe histopathological alterations in the brains of fish exposed to environmental contaminants. For example (65) reported marked damage to the brain architecture of common carp (*Cyprinus carpio*) following exposure to an organophosphate insecticide, including neuronal necrosis, intracellular edema, pyknotic nuclei, cytoplasmic vacuolization, neural cell degeneration, vascular dilation, and haemorrhagic lesions. The similarity between these pathological alterations and those observed in the present study suggests that oxidative stress and cellular degeneration represent common mechanisms underlying pollutant-induced neurotoxicity in fish.

The histopathological alterations observed in the brain may be attributed to the complex physiological functions of neural tissues and their high susceptibility to oxidative stress and toxic insults (66–68). Similar neurotoxic effects have been documented in fish exposed to various environmental contaminants, including extensive neuronal necrosis, loss of neuronal differentiation, cellular disorganization, and degeneration of brain tissues (69–72).

Several studies have demonstrated that exposure to B[a]P and other PAHs induces pronounced histopathological alterations in fish brain tissues. Reported lesions include neuronal degeneration, necrosis, vacuolization, edema, vascular congestion, haemorrhage, and disruption of normal brain architecture (20, 26, 30). In the present study, similar pathological changes were observed in the cerebellum of grass carp juvenile, including degeneration of cerebellar layers, neuropilar necrosis, disorganization of Purkinje cells, neuronal shrinkage, and increased vascularization. These findings further support previous observations that neural tissues are highly susceptible to PAH-induced toxicity. The observed neurotoxic effects may be related to mechanisms involving oxidative stress. Because the brain has high oxygen consumption and relatively limited antioxidant defenses, neuronal tissues are particularly susceptible to oxidative injury. These findings are consistent with previous reports suggesting that oxidative stress may contribute to B[a]P-induced neuronal damage and functional impairment (16, 20, 27).

The histological and histochemical observations confirmed the normal structural organization of the fish heart, consisting of three layers: the epicardium, myocardium, and endocardium. The epicardium is the outermost layer, composed of flattened epithelial cells and thin connective tissue (73). The myocardium, the thickest layer, consists of interconnected cardiomyocytes responsible for cardiac contraction and the production of biologically active molecules (73–75). The endocardium forms the innermost layer and comprises a thin endothelial lining supported by loose connective tissue.

In teleost fish, the atrial wall is generally thinner than the ventricular wall and plays a crucial role in maintaining cardiac function and regulating blood circulation (53, 75–77). Blood returning from the body enters the atrium through the sinus venosus, and atrial contraction facilitates the movement of blood into the ventricle for subsequent circulation. Similar cardiac architecture has been reported in several fish species, including *Echiichthys vipera*, *Oncorhynchus mykiss*, and *Salvelinus alpinus* (75). The normal cardiac morphology observed in the control group of the present study is therefore consistent with previous descriptions of teleost heart structure.

Histopathological examination in the present study revealed that exposure to B[a]P induced severe cardiac damage in grass carp (*Ctenopharyngodon idella*) juvenile. Atrial and ventricular tissues exhibited degeneration of the epicardial epithelium, edema, inflammatory cell infiltration, cardiomyocyte degeneration and necrosis, pyknotic nuclei, irregular trabecular organization, and widened lacunar spaces. Such structural abnormalities may impair cardiac contractility, disrupt blood circulation and oxygen transport, and ultimately compromise the overall physiological performance of exposed fish (78). Comparable cardiotoxic effects have been reported in fish exposed to pharmaceuticals, pesticides, hypoxic conditions, and other environmental contaminants that promote oxidative stress (79–81).

The observed cardiotoxicity may be associated with mechanisms involving oxidative stress. Previous studies have suggested that excessive ROS production can contribute to lipid peroxidation, mitochondrial dysfunction, membrane damage, inflammatory responses, and apoptotic pathways in cardiac tissues (82, 83). These pathological processes ultimately contribute to cellular degeneration, tissue necrosis, and impairment of normal cardiac function. Similar mechanisms have been proposed to explain PAH-induced cardiotoxicity in fish embryos and larvae, where oxidative stress plays a central role in cardiac malformations and functional abnormalities (29). Histochemical analysis further demonstrated alterations in cardiac polysaccharide distribution, as evidenced by variations in PAS-positive staining intensity among the different treatment groups. PAS-positive reactions indicate the presence of glycogen and other carbohydrate-rich macromolecules, including glycoproteins, within cardiac tissues. Changes in PAS staining patterns may reflect disturbances in cellular energy metabolism and structural integrity induced by B[a]P exposure. Similar observations have been reported in previous histochemical studies of fish cardiac tissues subjected to environmental stressors (84–87).

One limitation of the present study is that exposure was based on nominal B[a]P concentrations, and the actual concentrations in the exposure water were not analytically confirmed. Because B[a]P is a highly hydrophobic compound, some loss through adsorption to tank surfaces, suspended particles, or organic matter may have occurred during the exposure period despite daily preparation of fresh solutions and 50% water renewal. Consequently, the observed toxic effects should be interpreted with respect to the nominal exposure concentrations, and the precise dose-response relationship should be interpreted with caution. Future studies incorporating analytical measurements of B[a]P concentrations throughout the exposure period would provide more accurate exposure characterization and further strengthen the environmental relevance of these findings.

Collectively, the present findings demonstrate that nominal B[a]P exposure was associated with significant immunotoxic, neurotoxic, and cardiotoxic effects in juvenile grass carp. Although previous studies have identified oxidative stress as a major mechanism of B[a]P toxicity, the present study did not directly evaluate oxidative stress biomarkers or related molecular pathways. Therefore, the involvement of oxidative stress should be regarded as a biologically plausible explanation based on existing literature rather than a mechanism confirmed by the present data.

## 5 Conclusion

The present study showed that short-term exposure to nominal concentrations of B[a]P was associated with significant alterations in immune biomarkers, AChE activity, and histopathological and histochemical characteristics of the brain and heart in juvenile grass carp (*Ctenopharyngodon idella*). The observed changes in MPO activity, NO production, NBT reduction activity, and IgM levels suggest disruption of immune homeostasis, while the reduction in AChE activity together with the histopathological alterations in brain tissues is consistent with neurotoxic effects. Likewise, the structural and histochemical changes observed in cardiac tissues indicate cardiac tissue injury following B[a]P exposure. The integration of biochemical, histopathological, and histochemical endpoints provides a comprehensive assessment of the multi-organ responses associated with B[a]P exposure in juvenile grass carp. However, because oxidative stress, inflammatory pathways, apoptosis, and other molecular mechanisms were not directly investigated, the underlying mechanisms responsible for the observed alterations cannot be conclusively established. Furthermore, the exposure concentrations were nominal and were not analytically verified in water or fish tissues. Future studies incorporating environmentally relevant exposure concentrations, analytical verification of B[a]P residues, functional assessments, oxidative stress biomarkers, and molecular analyses are needed to further elucidate the toxicological mechanisms and ecological implications of B[a]P exposure in freshwater fish.

## Data Availability

The original contributions presented in the study are included in the article/supplementary material, further inquiries can be directed to the corresponding author/s.
